# Breast Cancer Affects Both the Hippocampus Volume and the Episodic Autobiographical Memory Retrieval

**DOI:** 10.1371/journal.pone.0025349

**Published:** 2011-10-10

**Authors:** Loretxu Bergouignan, Jean Pierre Lefranc, Marie Chupin, Nastassja Morel, Jean Philippe Spano, Philippe Fossati

**Affiliations:** 1 CNRS USR 3246, CR-ICM, Pitié-Salpêtrière Hospital, Paris, France; 2 Brain, Body and Self Laboratory, Department of Neuroscience, Karolinska Institute, Stockholm, Sweden; 3 Center for NeuroImaging Research, Pitié-Salpêtrière Hospital, Paris, France; 4 Department of Gynecologic Surgery, Pitié-Salpêtrière Hospital, Assistance Publique Hôpitaux de Paris, Paris, France; 5 Center for Research of the Institute of the Brain and of the Spinal Cord, Université Pierre et Marie Curie-Paris, UMR S875, Inserm U975, CNRS 7225, Paris, France; 6 Cognitive Neuropsychology and Functional Neuroanatomy of Human Memory, UMR S 923 INSERM, Caen Basse-Normandie University/EPHE/INSERM, Caen, France; 7 Department of Medical Oncology, Pitié-Salpêtrière Hospital, Assistance Publique-Hôpitaux de Paris, Paris, France; 8 Department of Psychiatry, Pitié-Salpêtrière Hospital, Assistance Publique-Hôpitaux de Paris, Université Pierre et Marie Curie, Paris, France; National Institute on Aging Intramural Research Program, United States of America

## Abstract

**Background:**

Neuroimaging studies show the hippocampus is a crucial node in the neural network supporting episodic autobiographical memory retrieval. Stress-related psychiatric disorders, namely Major Depression and Post Traumatic Stress Disorder (PTSD), are related to reduced hippocampus volume. However, this is not the case for remitted breast cancer patients with co-morbid stress-related psychiatric disorders. This exception may be due to the fact that, consequently to the cancer experience as such, this population might already be characterized by a reduced hippocampus with an episodic autobiographical memory deficit.

**Methodology:**

We scanned, with a 3T Siemens TRIO, 16 patients who had lived through a “standard experience of breast cancer” (breast cancer and a standard treatment in remission since 18 month) in the absence of any associated stress-related psychiatric or neurological disorder and 21 matched controls. We then assessed their episodic autobiographical memory retrieval ability.

**Principal Findings:**

Remitted breast cancer patients had both a significantly smaller hippocampus and a significant deficit in episodic autobiographical memory retrieval. The hippocampus atrophy was characterized by a smaller posterior hippocampus. The posterior hippocampus volume was intimately related to the ability to retrieve negative memories and to the past experience of breast cancer or not.

**Conclusions/Significance:**

These results provide two main findings: (1) we identify a new population with a specific reduction in posterior hippocampus volume that is independent of any psychiatric or neurological pathology; (2) we show the intimate relation of the posterior hippocampus to the ability to retrieve episodic autobiographical memories. These are significant findings as it is the first demonstration that indicates considerable long-term effects of living through the experience of breast cancer and shows very specific hippocampal atrophy with a functional deficit without any presence of psychiatric pathology.

## Introduction

It is generally accepted that damage to the hippocampus and related medial temporal lobe (MTL) structures leads to severe episodic autobiographical memory deficits [1], [2]. Moreover, functional brain imaging studies in healthy subjects demonstrated that the hippocampus is a crucial structure in the neural network supporting the access to episodic autobiographical memory [3]. Episodic autobiographical retrieval refers to the recall of personally relevant events acquired in a specific spatio-temporal context and characterized by an autonoetic state of consciousness [4]. This latter component enables conscious recollection of a personal event in its original encoding context and implies a mental time travel involving a vivid experience of remembering.

Animal studies have demonstrated that, beyond its role in memory, the hippocampus plays a key role in stress response [5]–[8]. Consistent with these animal studies, structural brain imaging studies in humans have repeatedly documented a 8 to 12% reduction in hippocampus volume in stress-related psychiatric disorders, that is Major Depression and Post Traumatic Stress Disorder (PTSD) [9]–[15]. Magnetic resonance imaging (MRI) studies investigated psychiatric stress-related pathology consequent to breast cancer [16]–[19]. Surprisingly, Inagaki et al. (2004) observed no difference in hippocampal volume between major depression after breast cancer, minor depression after breast cancer and remitted breast cancer patients without depression. Two predictions can emerge out of this result: Either the three patient groups had a hippocampus volume comparable to healthy people, or all three groups of patients had the same extent of hippocampus atrophy. The effect of breast cancer and its treatment as such on the hippocampal volume remains an open question. A working hypothesis is that the breast cancer experience as such is associated to a smaller hippocampus.

Interestingly, in addition to small hippocampus size, stress-related psychiatric disorders have been shown to be associated with episodic autobiographical memory retrieval deficits [20]–[24]. One previous study by Nilsson-Ihrfelt et al. (2004) investigated autobiographical memory retrieval in remitted breast cancer patients [25]. Even though in this study autobiographical memory was assessed solely for the specificity of the event -Autobiographical Memory Task, AMT [26]- without assessing episodic autobiographical retrieval components such as autonoetic consciousness [4], the results provided preliminary evidence of breast cancer patients deficits in autobiographical memories. In the current study we specifically tested the hypothesis of reduced hippocampus size, and associated episodic autobiographical memory retrieval deficits, in remitted breast cancer patients. In order to this, we employed a precise automatic hippocampus segmentation procedure to determine hippocampal volume, as well as a strict assessment of episodic autobiographical memory retrieval to define the nature of the functional memory deficit after having experienced breast cancer and its treatment. We aimed to answer the following questions: (i) Is the experience of breast cancer and its treatment associated to a reduced hippocampal volume? (ii) Is the experience of breast cancer and its treatment associated to episodic autobiographical memory deficits? (iii) Are the structural size of the hippocampus and the potential memory deficit linked?

## Results

### Episodic Autobiographical Memory assessment results

**Figure 1:**
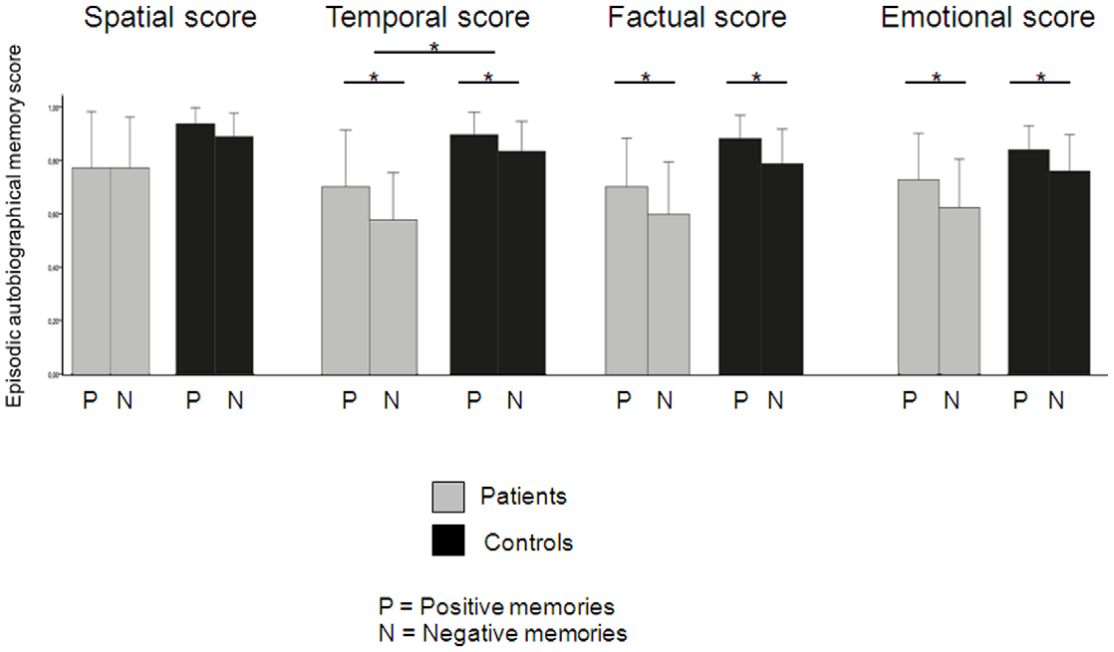
Behavioral analysis findings. Here are presented each group's emotional, factual, spatial and temporal Remember scores of positive autobiographical memories and negative autobiographical memories. The 2 (group: patients, controls) ×2 (valence: positive; negative) ×4 (episodic autobiographical memory score: emotional score, factual score, spactial score, temporal score) ANOVA showed a significant group effect and a significant valence effect but no interaction effect. Remitted breast cancer patients (who had no psychiatric or neurological disorder) had significantly lower episodic autobiographical memory retrieval than controls. Both groups had significantly higher episodic autobiographical memory retrieval for positive memories than for negative memories. *, *P*<0.05. See Table S1 for global Remember scores.

Remitted Breast cancer patients had significantly lower episodic autobiographical memory retrieval than controls (Figure 1). The ANOVA with the episodic autobiographical memory retrieval (Remember score for each aspect of the autobiographical memory -emotion, fact, time, space-) as the dependent variable revealed no aspect of the autobiographical memory effect (F = 1.716, df  =  35, p = 0.199), no interaction effects (F<1.22, df = 35, p>0.05), but a significant valence effect (F = 5.521, df = 35, p = 0.025) and a group effect (F = 4.216, df = 35, p = 0.048). Negative autobiographical events were significantly less episodically remembered than positive autobiographical events for both groups.

Remitted Breast cancer patients had 72.92% (+/−34.36) mean percentage of episodic autobiographical memory for positive events, and 64.58 (+/−30.5) for negative events, whereas control group had 89.29% (+/−15.62) for positive events and 82.14 (+/−20.12) for negative events.

### Hippocampus volume analyses

**Figure 2:**
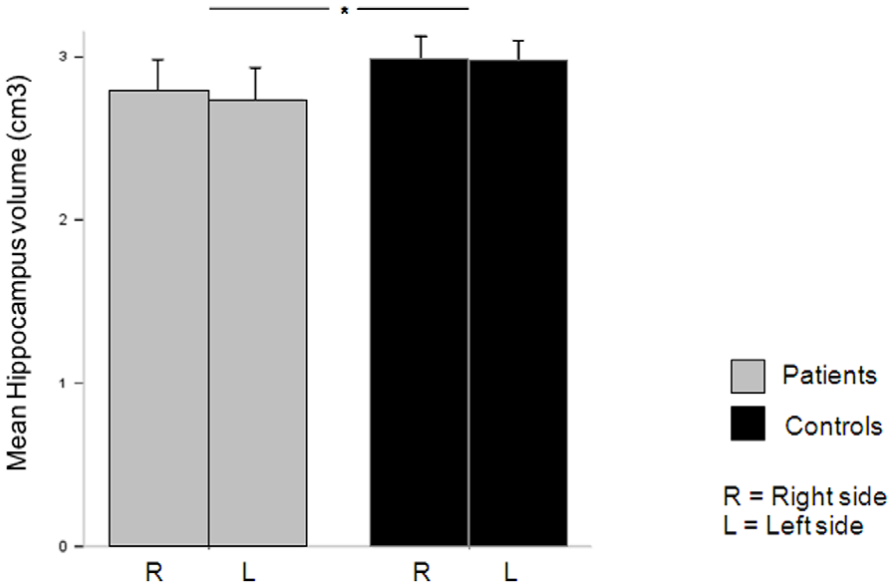
Hippocampus volumetric analysis findings. Mean of the cross-sectional volume measurements (corrected for TIV) for the left hippocampus and right hippocampus. Remitted breast cancer patients (who had no psychiatric or neurological disorder) had a significantly smaller hippocampus volume relative to controls. *, *P*<0.05.

Remitted breast cancer patients had significantly smaller hippocampi than controls (Figure 2).

All volume analyses that follow are corrected for the total intracranial volume (TIV). The ANOVA with the hippocampal volume (cm^3^) as dependent variable revealed no significant laterality effect (F = 0.026, df = 35, p = 0.451) or group×laterality interaction (F = 0.314, df = 35, p = 0.579) but a significant group effect (F = 4.702, df = 35, p = 0.037).

Left hippocampal volumes were 2. 88 cm^3^±0.40 (range: 2.48–3.28) for remitted breast cancer patients and 3.16 cm^3^±0.34 (range: 2.82–3.50) for age matched control participants. Right hippocampal volumes were 2.94 cm^3^±0.40 (range: 2.54–3.34) for remitted breast cancer patients and 3.17 cm^3^±0.39 (range: 2.82–3.56) for age matched control participants.

The mean hippocampal volume reduction in patients compared to controls was of 8% (8.8% on the left and 7.2% on the right). The t-test on TIV values showed no significant difference between groups (t = 1.627, df = 35, p = 0.113).

Posterior and anterior hippocampus are structurally and functionally very different [27]. Different pathologies can affect specific subparts of the hippocampus. In this standpoint, it has been shown stress-related psychiatric disorders are specifically related to reduced posterior hippocampus volume [9], [11]–[12], [28], we hypothesized that a breast cancer experience would also specifically affect this region of the hippocampus. The posterior hippocampus needs a very precise methodology for the analysis [9]. We thus segmented each subject's hippocampus using a precise automated segmentation. The t-test on the posterior hippocampus (mean of right and left posterior hippocampus volume) showed a significant group difference (t = −2.140, df = 35, p = 0.039). As hypothesised, remitted breast cancer patients had significantly smaller posterior hippocampus than controls. Posterior hippocampus volumes were 1.38 cm^3^±0.22 (range: 1.16–1.60) for remitted breast cancer patients and 1.52 cm^3^±0.16 (range: 1.36–1.68) for age matched control participants. The volume difference in the posterior hippocampus was of 11%.

We observed no significant difference in the anterior hippocampus (t = −1.74; p>0.05, see also Table 1).

**Table 1:** Details of hippocampal subpart volume in each population.

	Patients		Controls			
	Mean	S.D.	Mean	S.D.	p value	T
right Anterior hippocampus volume (cm3)	1.57	0.21	1.71	0.26	>0.05	−1.82
right Posterior hippocampus volume (cm3)	1.38	0.24	1.46	0.2	>0.05	−1.11
left Anterior hippocampus volume (cm3)	1.49	0.21	1.58	0.21	>0.05	−1.31
left Posterior hippocampus vilume (cm3)	1.39	0.28	1.58	0.18	0.016	−2.54

#### Episodic autobiographical memory and posterior hippocampal volume

The mixed regression model showed that the episodic autobiographical memory score was predicted by the group (t = −2.913; p = 0.006), the volume of the posterior hippocampus (t = −3.651; p = 0.001) and the interaction of group by volume of the posterior hippocampus (t = 3.340; p = 0.002).

## Discussion

The aim of our study was to assess the neural correlates of “breast cancer experience”. More precisely we were interested in hippocampus volume and potential episodic autobiographical memory deficit associated to a “breast cancer experience”. 1) We thus studied remitted breast cancer patients that had a “standard cancer treatment” and matched controls, 2) We measured their full hippocampi volume and their posterior hippocampus volume, 3) We assessed episodic autobiographical memory and 4) We looked at the regression analysis of the access to episodic autobiographical memory with the group and the volume of the posterior hippocampus as predictors.

The results of our study show that “breast cancer experience” is associated to a reduction in hippocampus volume. Specifically, as a group the remitted breast cancer patients of our study had an 8% reduction in global hippocampus volume, and a 11% reduction in posterior hippocampus volume compared to healthy controls. In addition, “breast cancer experience” is associated to a deficit in episodic autobiographical memory. The cancer patient group had 20% less access to episodic autobiographical memory than controls. However, both groups had significantly more access to positive memories than negative memories. The volume of the posterior hippocampus and the group were predicting the episodic autobiographical memory score.

### Breast cancer patients vs. control

We selected a population of patients with the aim of being representative of a “standard breast cancer experience”: We included patients in long term remission -within two years- after a “standard treatment” of breast cancer which included tumorectomy, chemotherapy and radiotherapy. While the period chosen is associated with a very high risk for psychiatric stress-related disorders [29], we specifically selected a population that did not suffer from any such disorder. Accordingly, on the contrary to previous studies we were interested neither in associated psychiatric stress-related disorders nor in the treatment effects [16]–[19], [30] considering the fact that the treatment is part of the “breast cancer experience”.

### Hippocampus volume

In this study we used an automatic hippocampus segmentation procedure that gives precise and systematic hippocampus segmentation, and we studied both whole hippocampi and posterior hippocampus volumes. It then allowed us to examine whether morphological changes were associated with the experience of breast cancer. Consistent with our hypothesis, the results show a reduced hippocampus volume in remitted breast cancer patients and more precisely a smaller posterior hippocampus. These findings are in line with the supposition that Inagaki et al. (2004) did not find any difference between patients with or without depression because the breast cancer experience per se affected the brain structure of their population, independently of the presence of depression or not. Our results are also in accordance with the assumption of stressful effect of breast cancer proposed by Nakano et al. (2002). Nakano et al. (2002) observed that the volume of the left hippocampus was significantly smaller (5%) in breast cancer patients with a history of distressing cancer-related memories compared to breast cancer patients without any such history. We have assessed exploratorily the history of distressing memories -intrusive memories- (in our patients but only eleven of the patients group sent back the questionnaire). In line with the stress induced hypothesis, when taking the subjects that experience higher intrusions (10 subjects: 6 patients, 4 controls) vs. subjects who experience lower intrusions (19 subjects: 5 patients, 14 controls), the posterior hippocampus and their interaction as predictive variables of episodic autobiographical memory score, we observed that the level of intrusion was a good predictor (t = −2.711; p = 0.011).

### Posterior hippocampus

Posterior and anterior hippocampus are structurally and functionally very different [27]. Different pathologies can affect specific subparts of the hippocampus. In this standpoint, it has been shown major depression and post-traumatic stress disorder are specifically related to reduced posterior hippocampus volume [9], [11]–[12], [28]; on the contrary, in schizophrenia, atrophy seems to mainly affect the anterior hippocampus [31]; also, mild cognitive impairment and/or Alzheimer disease begin with an atrophy of the anterior hippocampus [32].

Thus, the present study sheds new light on so far documented posterior hippocampus volume variability in humans, by identifying a new population with a specific reduction in posterior hippocampus volume that is independent of any psychiatric or neurological pathology (Table S1, for correlations between total hippocampal volume and hippocampus subparts volume). In this study, the specific anatomical abnormality found in the posterior hippocampus was also predictive of the episodic autobiographical memory retrieval score. It is the first study showing such a predictive value of the posterior hippocampus volume on episodic autobiographical memory retrieval ability.

As groups differ in MADRS one could suspect an influence of this difference in our results, but when taking the MADRS score as covariate, the ANCOVA showed again a significant group difference (F = 4.605; p = 0.039). Also, we need to underline that one has to be cautious in the interpretation of depression scales in cancer patients as symptoms such as fatigue and loss of energy, significant weight loss or gain, insomnia or hypersomnia are almost universal [33] in cancer patients even though major depression appears only for a minority of this population (about 20% of the population).

### Episodic autobiographical retrieval deficit

We assessed in the same population the episodic autobiographical memory retrieval to examine whether functional changes are associated to breast cancer experience. Remitted breast cancer patients had reduced access to episodic autobiographical memories. Using semantic and episodic tasks of autobiographical memory [34], Viskontas et al. (2000) found that patients with either right or left Medial Temporal Lobe (MTL) damage had a poorer memory for autobiographical events than controls [35]. Noulhiane et al. 2007, using our same task in epileptic patients, observed a deficiency in access to episodic autobiographical memories after MTL resection [1]. These studies rely on lesions of whole hippocampus or larger medial temporal lobe, in our study, we observed a very specific structural volume damage, namely to the posterior hippocampus and still showed a deficit in episodic autobiographical memory.

Since we used a cross-sectional design, we could not confirm any causality between hippocampal changes, functional changes, and a history of breast cancer experience. It may be that the differences existed before the experience of cancer, or that a reduction of volume occurred during the cancer experience in itself. Longitudinal studies in this specific population are needed to assess this further.

### Positive bias maintained

A positive bias is an essential aspect of healthy individuals' strength [36]. Unlike psychiatric patients with stress related disorder [24], [37], our remitted breast cancer patients with no neurological or psychiatric co-morbidity show a positive bias in their episodic autobiographical memory retrieval. Both patients and controls showed significantly higher episodic autobiographical score for positive memories than for negative memories. Smaller hippocampus and less episodic autobiographical memory access is thus not unique to stress related psychiatric pathologies; it can be present in an healthy population that experienced breast cancer but maintain a positive bias on their past.

### Limits of the study

Our study has some limitations: a small sample of patients and a cross-sectional design. Large scale longitudinal studies evaluating the link between potential posterior hippocampal atrophy and episodic autobiographical memory performances of breast cancer patients are needed. Also, we did not assess the effect of each specific treatment, concerning the results, we thus do not exclude the influence of the treatment itself even though first studies on chemotherapy (which is the most invasive part of the treatment) show no adverse effect on hippocampal volume of breast cancer survivors [30]. Four of our patient group had an hormonotherapy treatment. One could hypothesis an effect of hormonotherapy in our results; however, when using the presence of hormonotherapy as covariate in the analysis, we observed a very significant between group difference in the hippocampus volume (F = 9.968, p = 0.003).

### Cumulative negative/stressful event effect hypothesis

It is well known that diagnosis and treatment of breast cancer imply experiencing short and long-term cumulative stressful life events: Accepting the threat of death, physical changes, chemotherapy and radiotherapy [29], [38].

Over the past decades, a large body of research has investigated the effects of stress-related psychiatric disorders on the brain. One line of research has focused on structural brain imaging techniques, repeatedly documenting that patients suffering from Major Depression or PTSD have a reduced (posterior) hippocampus [9]–[15]. Another line of research has instead focused on the cognitive and psychological consequences of this disorder, showing that Major Depression and PTSD suffer from autobiographical memory deficits [20]–[24]. However, despite these robust findings, so far no studies have systematically tried to link the two lines of research by investigating the link between the alteration of hippocampus structure and autobiographical memory performance. In the present study, even in the absence of psychiatric co-morbidity, we found experience of breast cancer to be associated with a smaller posterior hippocampus and deficits of episodic autobiographical memory, which corresponds to very similar discrepancy as observed in stress-related psychiatric disorders. The common point of breast cancer experience, Major Depression and PTSD is their exposure to negative events with heavy and/or cumulative stress. We thus consider that the smaller posterior hippocampus and the global deficit of episodic autobiographical memory would reflect the effect of exposure to negative events per se and not a specific effect of psychopathology. As underlined above, unlike stress related psychiatric pathologies [24], [37], our remitted breast cancer patients showed the same positive bias as healthy controls: the lack of positive bias may thus be more specific to psychiatric stress related pathologies.

In regard to the neuro-chemical reactions to cumulative stress it has been shown that acquisition of a conditioned contextual fear response is dependent on *N*-methyl-D-asparate (NMDA) receptor-mediated mechanisms in the dorsal hippocampus of animals (which corresponds to the posterior hippocampus in humans). In addition, it has been found that contextual learning increases levels of brain-derived neurotrophic factor in small mammals' dorsal hippocampus, whereas exposure to stress (mammals artificially stressed or injection of IL-1B) reduces it [39], [40]. Also, in humans an effect of stress and glucocorticoids in memory processes associated to the hippocampus volume has been recently shown by Coluccia et al. [41]. We thus further speculate the predictive value of the posterior hippocampus and the past experience of breast cancer on the episodic autobiographical memory deficit reflect this effect of exposure to stressful events on the posterior hippocampus.

### Conclusion

This study demonstrates for the first time that the breast cancer experience does not affect only the life of the patients, but also their brain. Remitted breast cancer patients had both a smaller posterior hippocampus and less ability to retrieve episodic autobiographical memories. It is also the first study to assess the specific effect of smaller posterior hippocampus on the access of episodic autobiographical memory. We show the posterior hippocampus volume is intimately related to the ability to access episodic autobiographical memories. The presence of a past experience of breast cancer is also intimately related to the ability to access episodic autobiographical memories. This study is thus in accordance with the suggestion that the posterior hippocampus is affected by exposure to negative events with heavy or cumulative stress. It seems that there is a capacity for local plastic change in the structure of the healthy adult human brain in response to breast cancer experience without a necessary change in the positive bias.

## Materials and Methods

### Ethics Statement

The current study was approved by the institutional review board as well as the ethics committee of the Salpêtrière hospital, and written informed consent was obtained from all participants.

### Subjects

Patient inclusion criteria were: (i) Age ranging between 18 and 55 years. We choose this age range because young patients typically suffer from significantly higher psychic distress than elderly patients [42]; (ii) Treatment of cancer by tumorectomy, chemotherapy and radiotherapy; (iii) 18 to 36 months post radiotherapy treatment as this period is known to be a vulnerability period for stress-related disorders (patients currently in hormono-therapy were included); (iv) No cancer relapse; (v) No secondary cancer to the breast cancer; (vi) No major depressive disorder fulfilling the criteria of the DSM-IV (MADRS <10) before, during or after breast cancer; (vii) No Post-traumatic Stress Disorder (PTSD) before, during or after breast cancer; (viii) No psychiatric or neurological disease; (ix) No past or present use of antidepressants or anxiolytics.

Forty-eight patients treated in Salpêtrière hospital between 2004 and 2006, with tumorectomy and chemotherapy for unilateral breast cancer diagnosis, were preselected. All participants were initially sent an invitation letter by their surgeon of the mammal surgery unit of the Salpetriere's Hospital (Paris). Subsequently, participants were contacted to schedule an appointment for the clinical assessment, the episodic autobiographical memory assessment and the MRI scanning session.

Thirty-two patients of the pre-selected group were excluded: for breast *(5)* or other part of the body *(3)* tumor recidive, for past or present depression *(6)* or use of antidepressant drugs *(2)*, for having a comorbidity with a neurological pathology *(2)*, for not speaking fluently French *(1)* and for having exclusion criteria for MRI scanning *(2)*. Six patients did not want to participate to the study because of being afraid of the scanning session. Five could not participate because of living abroad *(3)* or not allowed to take days off in their work *(2)*. In total, sixteen women in remission from breast cancer without co-morbidity were included. Among the twenty-eight contacted healthy participants (female, age between 40–55), seven were excluded: for past depression *(1)*, for eating psychiatric disorder *(1)* and for exclusion criteria for MRI scanning *(5)*. In total twenty-one healthy controls were included. No controls had had any serious physical disease.

Thus, sixteen women in remission from breast cancer and twenty-one healthy controls participated in this study. None of the patients or controls had any history of psychiatric or neurological disorder and all were fluent in French.

### Clinical assessment and Behavioural analyses

Clinical and demographic characteristics of the participants are displayed in Table 2.

**Table 2:** Clinical and demographic characteristics of the participants.

	Patients		Controls			
	Mean	S.D.	Mean	S.D.	p value	T
Age	48.73	4.95	47.68	5.313	>0.05	0.607
Years of education	13.91	2.39	13.38	2.849	>0.05	0.792
WAIS-R	104.27	11.49	103.41	12.462	>0.05	0.212
Age at the beginning of symptoms	44.91	4.35				
Age at diagnosis	45.27	3.37				
Mean duration of disease (month)	9.5	3.37				
Tumorectomy (number of weeks after diagnosis)	7.5	7.17				
Chemortherapy lasting (number of week)	16.4	7.41				
Radiotherapy lasting (number of week)	5.8	3.74				
Remission lasting (month)	39.27	16.61				
MADRS	3.25	2.59	1.62	1.936	0.035	2.192
HAD-A	6.36	2.38	5.84	3.5	>0.05	0.665
HAD-D	3.91	2.47	2.11	1.595	0.02	2.439

Participants were screened for DSM-IV Axis I disorders with the Mini-International Neuropsychiatric Interview [43] and for borderline and schizotypal personality disorder with the structured clinical interview -SCID- [44]. Information about the onset of breast cancer, duration of the treatment, and length of remission were recorded for each patient (patients were considered as remitted if their radiotherapy had been completed 18 to 36 months prior to the study).

Participants' verbal IQ was assessed with the revised verbal Wechsler Adult Intelligence Scale (verbal WAIS-R).

### Episodic Autobiographical Memory assessment

For the assessment of autobiographical memory we used the TEMPau task, a semi-structured interview developed by Piolino et al. [45]–[47], [24], [37], [1]. Similar to the Autobiographical Memory Task developed by Williams & Broadbent [26], the TEMPau consists of asking the participants to retrieve specific events located in time and space, which occurred once and lasted less than 1 day. However, while the AMT uses a time limit, the TEMPau does not. The TEMPau version we used assesses the subjective state of consciousness, and the First/Third person perspective in three life periods: Prior to the last 5 years (the ‘no cancer’ period), the last 5 years except for the last 12 months (the ‘cancer’ period), and the last 12 months (the ‘past history of cancer’ period). Each life-period was assessed for two positive and two negative events. Life-periods were randomly presented. The emotional valence (positive or negative) and the life-period of the event were the only cues provided to the participants. No word-cue was given to ensure that the participants began the retrieval from the most general level of the Conway's Self Memory System (lifetime periods) [48]. Immediately after each retrieval, the visual perspective was assessed by the First person perspective vs. Third person perspective procedure [49]–[50]. The participants' response could be of 3 types: a) A *First person perspective response* (if the content of their memories was in the same visual perspective as in the original event); b) a *Third person perspective response* (if they saw themselves in the event from the perspective of an external observer); c) a *Both perspective response* (if they had a conjunction of the two visual perspectives in the retrieved scene). If participants gave this latter response, they were asked to additionally provide the dominant visual perspective.

For the assessment of the subjective state of consciousness (i.e. the episodic access to autobiographical memories) we used the remember/know paradigm [51]. A *Remember response* was defined as the ability to mentally relive specific aspects such as perceptions, thoughts or feelings that were experienced at the time of the event (thus, as the ability to have access to the emotional, factual, spatial, or temporal context of the event). The participants were asked to give details aloud to ensure that they were using Remember responses properly. A *Know response* was defined as simply knowing what emotions they had, what happened, where and when, but without this knowledge being accompanied by any conscious recollection or access to the context of the encoding. A *Guess response* corresponded to aspects of the event that were neither consciously recollected nor simply known, but guessed.

### Episodic Autobiographical Memory scores

The Remember score defines the number of justified Remember responses divided by the sum of justified Remember and Know responses (Remember responses without associated details were discarded from the analysis).

The First person perspective score defines the proportion of First person perspective dominance.

#### Statistical analysis

A repeated measure ANOVA, with groups (patients versus controls) as a two-level- between subject factor, valence (positive or negative) as within-subject factor and Remember score (R) according to memory components (R emotional, R factual, R spatial, R temporal) as the dependent variable was carried out to assess the ability to retrieve episodic autobiographical memories.

For a secondary and more exploratory assessment of visual perspective in autobiographical memories, a repeated measure ANOVA, with groups (patients versus controls) as a two-level- between subject factor, valence (positive or negative) as within-subject factor and First person perspective score as the dependent variable was carried out to assess the amount of episodic autobiographical memories viewed in First person perspective during retrieval.

### MRI data acquisition

MRI scanning took place during the same week as the Episodic Autobiographical Memory assessment. High-resolution three-dimensional T_1_-weighted images were acquired with a 3 Tesla Siemens TRIO 32-channel TIM scanner (Siemens Medical Solutions, Erlangen, Germany), with a 12-channel head coil. We use a sagital acquisition of a 3D fast gradient echo inversion recovery sequence, with inversion time: 400 ms, repetition time: 2,300 ms, echo time: 4.18 ms, bandwidth: 150 Hz, flip angle: 9°, matrix: 256×256, field of view: 220×220 mm, voxel size: 1×1×1 mm^3^.

### Whole-brain volumes

The Total Intracranial Volume (TIV) was computed as the sum of Grey Matter (GM), White Matter (WM) and Cerebrospinal Fluid (CSF) volumes, derived from the segmentation toolbox of Statistical Parametric Mapping 5 (SPM5) (Wellcome Department of Cognitive Neurology, Institute of Neurology, London, UK, http://www.fil.ion.ucl.ac.uk/spm/).

### Full hippocampus and posterior hippocampus analysis using SACHA

We segmented each subject's hippocampus using the automated segmentation SACHA software [52]–[54], within the Brainvisa image analysis platform (Institut Fédératif de Recherche de Neuroimagerie, IFR 49, http://www.brainvisa.info/). The program allows one to determine the volume of each hippocampus. After manually entering the border between the head and the body of the hippocampus, it also allows one to exactly determine the volumes of the anterior (head) and posterior (body and tail) hippocampus.

**Figure 3:**
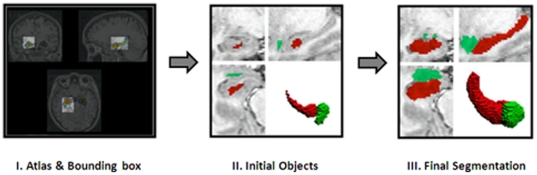
Fully automatic segmentation of the hippocampus with SACHA. Initialization (I.) extraction of the bounding boxes from the probabilistic atlases; (II.) extraction of the initial objects from each probabilistic atlas through conditional pruning; (III.) Obtained final segmentation. This method gives equivalent hippocampus segmentations as a manual segmentation (Bergouignan *et al.*, 2009; Chupin *et al.*, 2009). It gives global volumes of the hippocampus of each side (right and left). It also gives subparts volumes of the hippocampus (posterior subpart -including body and tail- and anterior subpart) after a manual insertion of the limit of the head.

SACHA extracts the hippocampus from a native scan according to the protocol described in Chupin et al. [52]–[54]. The extraction includes the fimbria, alveus, dentate gyrus, cornu ammonis, whereas the subiculum is excluded. The SACHA software relies on region deformation, introducing a competition between the hippocampus and the amygdala. It gives the volume of both regions, but in the current study we only took hippocampal volumes into account. First, an extraction of a bounding box is done from probabilistic atlases. The bounding box is defined around the amygdalo-hippocampal complex. A conditional pruning gives the initial objects composed of the region with maximal probability in each probability map. These two initial objects constitute the starting point of the deformation process. During the deformation, the algorithm automatically aggregates voxels and converges to the segmentation of the hippocampus and amygdala structures (Figure 3).

The whole procedure takes about 10 minutes for each side. Measurements were controlled by a trained rater (LB) who was blind to group and clinical information.

The SACHA algorithm has been validated only on native data. We used this standard version and obtained non-normalised hippocampal segmentations. We thus took into account brain inter-subject variability normalizing each obtained volume with the TIV.

#### Statistical analysis

A repeated measure ANOVA was used to test for differences between patients and controls, with one two-level-between-subject factor (group: patients, controls), and one two-level-within-subject factor (laterality: left, right).

As we were particularly interested in the posterior hippocampus we added an independent sample t-test on the posterior hippocampus.

In order to assess the link between the episodic autobiographical retrieval capacity; the volume of posterior hippocampus and the past experience of breast cancer, we made a mixed model regression analysis on the global episodic autobiographical memory score with the group (patients, controls); the volume of the posterior hippocampus and their interactions as predictors.

## Supporting Information

Table S1 **Correlations between total hippocampal volume and hippocampus subparts volume and correlation between global episodic autobiographical memory score and episodic autobiographical memory components sub-scores.**
(DOCX)
