# Supplementary material for: Breast Cancer Affects Both the Hippocampus Volume and the Episodic Autobiographical Memory Retrieval
Source: PLoS One. 2011 Oct 10;6(10):e25349. doi: 10.1371/journal.pone.0025349 (PMC3189914; doi:10.1371/journal.pone.0025349)
Supplement: Table S1 — Correlations between total hippocampal volume and hippocampus subparts volume and correlation between global episodic autobiographical memory score and episodic autobiographical memory components sub-scores. (DOCX) [file pone.0025349.s001.docx]

Table S1

| **Hippocampus volume** |  |  |
| --- | --- | --- |
| *Left Hippocampus volume* |  |  |
| correlation with left posterior hippocampus volume | R=0.825 | p<0.001 |
| correlation with left anterior hippocampus volume | R=0.872 | p<0.001 |
| *Right Hippocampus volume* |  |  |
| correlation with right posterior hippocampus volume | R=0.681 | p<0.001 |
| correlation with right anterior hippocampus volume | R=0.559 | p<0.001 |
| **Episodic Autobiographical memory global score** |  |  |
| *Positive Episodic Autobiographical memory global score* |  |  |
| correlation with positive emotional score | R=0.916 | p<0.001 |
| correlation with positive factual score | R=0.927 | p<0.001 |
| correlation with positive spatial score | R=0.914 | p<0.001 |
| correlation with positive temporal score | R=0.937 | p<0.001 |
| *Negative Episodic Autobiographical memory global score* |  |  |
| correlation with negative emotional score | R=0.860 | p<0.001 |
| correlation with negative factual score | R=0.897 | p<0.001 |
| correlation with negative spatial score | R=0.789 | p<0.001 |
| correlation with negative temporal score | R=0.811 | p<0.001 |
